# Diagnosis Value of Colposcope Combined with Serum Squamous Cell Carcinoma Antigen, Carbohydrate Antigen 125, and Carcinoembryonic Antigen for Moderate to Advanced Cervical Cancer Patients Treated with Modified Fuzheng Peiyuan Decoction

**DOI:** 10.1155/2021/4355805

**Published:** 2021-12-31

**Authors:** Huijuan Meng, Yulan Zhang, Youguo Chen

**Affiliations:** ^1^The Department of Obstetrics and Gynecology, The First Affiliated Hospital of Soochow University, Suzhou 215000, Jiangsu, China; ^2^The Department of Obstetrics and Gynecology, Suzhou Hospital of Traditional Chinese Medicine, Suzhou 215003, Jiangsu, China

## Abstract

**Objective:**

To explore the diagnosis value of colposcope combined with serum squamous cell carcinoma antigen (SCC-Ag), carbohydrate antigen 125 (CA125), and carcinoembryonic antigen (CEA) for moderate to advanced cervical cancer patients treated with modified Fuzheng Peiyuan decoction.

**Methods:**

The clinical data of 43 moderate to advanced cervical cancer patients treated in Suzhou Hospital of Traditional Chinese Medicine from July 2018 to July 2019 were selected for the retrospective analysis, and 43 healthy women undergoing physical examination in our medical center in the same period were selected as the control group. The cervical cancer patients accepted the modified Fuzheng Peiyuan decoction treatment, the detection of SCC-Ag, CA125, and CEA and colposcope examination were performed to all research subjects, and the changes in indicators such as KPS scores and lesion perfusion parameters in cervical cancer patients before and after treatment were monitored, so as to analysis the clinical diagnosis value of combined diagnosis in treated patients.

**Results:**

After treatment, the mean KPS scores were greatly higher and various blood perfusion parameters of lesions and serum SCC-Ag, CA125, and CEA levels were remarkably lower than before (*P* value <0.001 for all); the area under the curve of combined test was significantly larger than that of single test, and the sensitivity and specificity of the combined test were the highest; and after medication, the total incidence rate of toxic and side effects was 11.63%.

**Conclusion:**

Fuzheng Peiyuan decoction has significant effect in treating moderate to advanced cervical cancer, and colposcope combined with serum test presents more accurate and credible diagnosis results and has great significance for future treatment, which shall be promoted and applied.

## 1. Introduction

Cervical cancer is the most common malignant disease in women. There were 527,000 new cervical cancer cases in 2018; and every year in China, about 30,000 patients die of the disease and the maximum number of new cases can reach 12,000 [1, 2]. Currently, the methods for early screening and diagnosis of cervical cancer are still immature, resulting in a large proportion of patients already in middle or advanced stage at the time of presentation, thereby missing the optimal timing of surgical resection. In the late stage of cervical cancer, because the tumor invades adjacent tissues or organs, patients will suffer from urgent micturition, frequent micturition, and sensation of anal bulge [3], as well as complications such as ureteral obstruction and damage to renal function, seriously harming life and health. Cisplatin is a common chemotherapeutic agent used in the current treatment of moderate to advanced ovarian cancer [4], which can effectively kill tumor cells, but at the same time cause certain adverse reactions including myelosuppression and immune function impairment that are intolerable for patients [5]. Traditional Chinese medicine (TCM) has accumulated a large amount of experience in the treatment of gynecologic tumor diseases, and modern pharmacological studies have confirmed [6] the enriched astraglan component in Fuzheng Peiyuan decoction can enhance immunity and inhibit the pathogenic activity of virus, which has been demonstrated in the treatment of nonsmall cell lung cancer [7]. Colposcopy is a medical device used in the clinic to view the changes in the vagina, vulvar tissue, and cervical epithelium, which can assist in the localization and improve the positive rate of biopsies [8]. However, it also has some limitations, for instance, in elderly patients with stenosis of the vagina, colposcopy cannot achieve a good result in examining cervical adhesion, and only the 1-2 layers of epithelium on the surface can be viewed, but not the deep infiltrating carcinomas [9]. Therefore, an examination method with high diagnostic efficacy that can precisely grasp the lesion condition of such patients is necessary to provide them with highly effective treatment regimens. Serum marker detection is widely used in clinical practice in recent years for the diagnosis of cervical cancer and assists tumor screening, diagnosis, and efficacy evaluation. Based on this, the clinical diagnosis value of colposcope combined with serum SCC-Ag, CA125, and CEA for patients with moderate to advanced cervical cancer treated with modified Fuzheng Peiyuan decoction was explored herein.

## 2. Data and Methods

### 2.1. General Information

The clinical data of 43 moderate to advanced cervical cancer patients treated in Suzhou Hospital of Traditional Chinese Medicine from July 2018 to July 2019 were selected for the retrospective analysis, and 43 healthy women undergoing physical examination in our medical center at the same period were selected as the control group.

### 2.2. Inclusion and Exclusion Criteria

#### 2.2.1. Inclusion Criteria

① The patients met the diagnosis standards of cervical cancer in three-step diagnostic technique for cervical lesions [10] and were diagnosed after histopathological inspections, and the clinical manifestations included frequent micturition, urgent micturition, anal swelling, and contact bleeding; ② according to the international clinical staging criteria passed by the International Society of Obstetrics and Gynecology, the clinical stage was III-IV [11], and the patients did not receive chemoradiotherapy intervention before recruitment; and ③ the study met the World Medical Association Declaration of Helsinki [12] and was approved by the ethics committee of the Suzhou Hospital of Traditional Chinese Medicine.

#### 2.2.2. Exclusion Criteria

① The patients had immune or coagulation dysfunction; ② the patients took anticancer TCM preparation, immunomodulator, etc., four weeks before recruitment; and ③ the patients were allergic to the decoction applied in the study.

### 2.3. Treatment and Test Methods

Fuzheng Peiyuan decoction treatment was performed to patients with moderate to advanced cervical cancer, and the decoction contained 15 g of solomonseal rhizome, 15 g of ganoderma powder, 15 g of wild Chinese wolfberry fruit, 15 g of danshen root, 15 g of Chinese angelica, 12 g of epimedium herb, 12 g of medicinal cyathula root, 20 g of dodder seed, 20 g of prepared rehmannia root, and 30 g of Mongolian milkvetch root. The herbs were decocted with water to 300 ml as one dose and taken in two split times daily for 2 months.

The research objects of the two groups received colposcope and serum indicator examinations.

#### 2.3.1. Colposcope Examination

The colposcope examination was performed to patients at a later date after the end of menstruation. Twenty-four hours before examination, vaginal administration, vaginal douche, and sexual life were prohibited. During examination, the patients took the bladder lithotomy position and relaxed, the vaginal speculum was put after routine vulva disinfection, 3% acetic acid solution was used to apply to the cervix uteri, the colposcope (model: HD902; manufacturer: Wuhan Yimei Medical Equipment Co., Ltd.) after adjusting the focal length of the probe, the cervix uteri image was enlarged on the display screen for observation, when the best image was obtained, normal saline was used to flush and clean the secretions, and after staining by the compound iodine solution, the condition of cervix uteri was observed under colposcope.

#### 2.3.2. Serum Indicator Measurement

Before and after treatment, 5 ml of fasting venous blood was drawn from the patients before and after treatment, the serum was separated after procoagulant processing, the content of SCC-Ag in serum was measured by chemiluminescent microparticle immunoassay, and the content of CA125 and CEA was measured by enzyme-linked immunosorbent assay (ELISA). The measurement was operated according to the specification on the kits, which were purchased from Shanghai Enzyme-Linked Biotechnology Co., Ltd.

### 2.4. Observation Indicators

#### 2.4.1. Physical Strength

The physical strength of patients before and after treatment was evaluated with the Karnofsky scores (KPS) [13], and on a scale of 0–100 points, higher scores indicated better physical strength.

#### 2.4.2. Lesion Perfusion Parameters

The quantitative perfusion parameters, including the maximum intensity (IMAX), rise time (RT), time-to-peak (TTP), and mean transit time (mTT) at the cervical lesions in patients before and after treatment were measured by the US guided examination with DW-TB color Doppler ultrasonic diagnostic apparatus (manufacturer: Shanghai Mingyuan Industry Company Ltd.).

#### 2.4.3. Serum Indicators

On the next day that the cervical cancer patients were admitted to the hospital and the day that the healthy individuals received physical examination, 5 ml of their fasting venous blood was drawn in the morning to separate the serum after centrifugation and then measure the contents of SCC-Ag, CA125, and CEA in the serum samples by ELISA method in strict accordance with the operation steps in the instructions of kits (manufacturer: Yilaisa Biotechnology Co., Ltd. (Jiangsu, China)).

### 2.5. Statistical Methods

In this study, the statistical analysis and processing of experimental data were conducted with SPSS 23.0, the picture drawing software was GraphPad Prism 7 (GraphPad Software, San Diego, USA), the enumeration data were examined by *t*-test and expressed by mean ± SD, differences were considered statistically significant at *P* < 0.05, and with Mann–Whitney *U* test, the clinical diagnosis value was analyzed by the area under the receiver operating characteristic curve (ROC curve).

## 3. Results

### 3.1. Comparison of KPS Scores before and after Treatment

**Figure 1:**
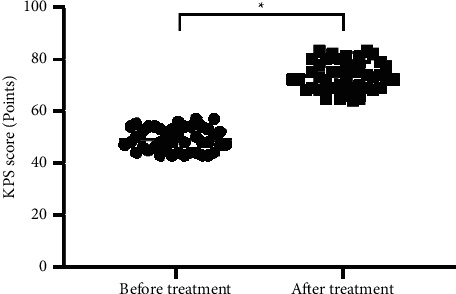
Comparison of KPS scores before and after treatment (mean ± SD). Note: the horizontal axis denotes before and after treatment, and the vertical axis denotes the KPS score (points); before and after treatment, the patients' mean KPS scores were (49.07 ± 4.35) and (73.42 ± 5.47), respectively; and ^*∗*^ indicates significant difference in the mean KPS scores before and after treatment (*t* = 22.847, *P* < 0.001).

After treatment, the patients' mean KPS scores were greatly higher than before (*P* < 0.001). See Figure 1.

### 3.2. Comparison of Blood Perfusion Parameters of Lesion before and after Treatment

After treatment, various blood perfusion parameters of lesion were significantly lower than before (*P* < 0.001). See Table 1.

### 3.3. Comparison of Changes in Serum SCC-Ag, CA125, and CEA Levels before and after Treatment

After treatment, the serum SCC-Ag, CA125, and CEA levels were significantly lower than before (*P* < 0.001). See Table 2.

### 3.4. Analysis on Clinical Diagnosis Value of Combined Test for Patients after Treatment

**Figure 2:**
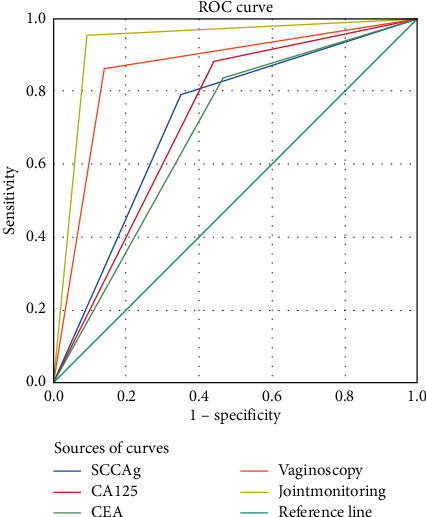
Analysis on clinical diagnosis value of combined test for patients after treatment.

Analysis on clinical diagnosis value of combined test for patients after treatment is shown in Figure 2.

### 3.5. Comparison of Area, Standard Error, Progressive Significance, and 95% Confidence Interval of Various Indicators

The results of combined test were higher than various single test. See Table 3.

### 3.6. Comparison of Positive Rate, Sensitivity, and Specificity of Various Indicators

The sensitivity and specificity of combined test were the highest. See Table 4.

### 3.7. Toxic and Side Effects

By observing the occurrence of toxic and side effects in 43 patients during treatment, it was found that the total incidence rate was 11.63% (5/43), including 2 cases with gastrointestinal reaction, 1 case with liver and kidney dysfunction, and 2 cases with platelet reduction. The patients with toxic and side effects were recovered after administration withdrawal.

## 4. Discussion

Cervical cancer is a common female reproductive disease with nontypical symptoms such as vaginal bleeding [14] and bloody leukorrhea that are also manifested in other gynecological inflammatory diseases, and combined with poor health awareness and fear of disease, most patients are diagnosed when they are in middle to late stages of the disease [15]. Malignant tumors can metastasize to the lymph and abdomen, and even distant sites such as the lung, pleura, and liver, seriously endangering women's life and health [16, 17]. Cisplatin, as a common drug for the treatment of cervical cancer, exerts the therapeutic effect by inhibiting the mitosis of tumor cells, but long-term use increases the drug resistance of tumor cells, which can produce severe toxic and side effects and then result in unbearable effects for patients [14, 18]. TCM has accumulated a lot of experience in the treatment of cervical cancer, and modified Fuzheng Peiyuan decoction is a TCM preparation for strengthening vital qi, which mainly contains Mongolian milkvetch root, dodder seed, and prepared rehmannia root. Among them, Mongolian milkvetch root has the effects including diuresis and elimination of toxicant, and invigorating qi for consolidating body resistance, dodder seed can strengthen and nourish liver and kidney, strengthen kidney to stop emission, and arrest polyuria, and prepared rehmannia root works well in nourishing yin and blood as well as benefiting blood and vessels. The formula is able to invigorate spleen, kidney, qi, and yin, which can play a role in inhibiting cancer cell proliferation and improving the condition [19].

With the intensive study of serum biochemical indicators, it has been found that SCC-Ag exists in the cytoplasm of squamous cell carcinoma of the uterus, cervix, lung, etc., and is used in the auxiliary diagnosis for these cancers. The SCC-Ag content in the serum of patients with advanced cervical cancer is higher than that in patients of the early stage, indicating that it is increasing with the malignant progression of cervical lesions and the advancement of specific stage [20]. CA125 and CEA are two common serum tumor markers, the content of which is significantly elevated in many cancers, such as epithelial ovarian cancer, carcinoma of fallopian tube, and lung cancer, and related reports have indicated [21, 22] that high levels of CA125 and CEA have important reference values in the diagnosis and prognosis of cervical cancer. The results of this study showed that patients exhibited significant decreases in all serum markers after treatment with the modified Fuzheng Peiyuan decoction, indicating that this TCM formula could reduce serum tumor marker levels in patients with advanced cervical cancer and was important for improving outcomes.

As a noninvasive diagnostic modality, colposcopy can observe color, vascular structure, and morphological structure of the abnormal parts of the cervix in patients in many ways, but not the lesions in the cervical canal, resulting in poor diagnostic results. Colposcopy combined with serum detection can significantly improve the pathological diagnosis rate of cervical cancer, because serum detection is convenient and can directly reflect the number, spread, and apoptosis of cancer cells [23]. Colposcopy is an imaging means currently used to screen for precancerous cervical lesions, which can amplify the image of the cervix and vagina mucosa, enable more precise visualization of tiny lesions, vascular tissue, and suspicious areas on the surface of the cervix that are not visible to the naked eye, and localize the biopsy to improve test accuracy. Therefore, combining colposcopy with serum detection can eliminate problems such as unnecessary treatment for false positive patients and ensure safety, reliability, and strong operability, which is suitable for clinical diagnosis and efficacy determination [24]. In this study, the efficacy of Fuzheng Peiyuan decoction in the treatment of moderate to advanced cervical cancer was evaluated by the combined test, and by plotting the ROC curve, it was found that combined test had an area under the curve larger than each single test, and high sensitivity, and specificity, and therefore it might serve as an effective diagnostic modality to objectively evaluate the treatment effect of cervical cancer patients. This study has some deficiencies. The selected patients were all treated in our hospital, so the source of cases lacked diversification; in addition, follow-up visits for the long-term efficacy of patients were not carried out. To sum up, the initial conclusion obtained in this study shall be perfected by more researches in the future.

In conclusion, Fuzheng Peiyuan decoction has significant effect in treating moderate to advanced cervical cancer, and colposcope combined with serum test presents more accurate and credible diagnosis results and has great significance for future treatment, which shall be promoted and applied.

## Figures and Tables

**Table 1:** Comparison of blood perfusion parameters of lesion before and after treatment (mean ± SD).

Time	IMAX	RT (s)	TTP (s)	mTT (s)
Before treatment	25.17 ± 6.18	20.16 ± 3.17	34.27 ± 8.52	43.18 ± 4.92
After treatment	14.57 ± 3.08	13.27 ± 2.76	25.15 ± 7.58	29.71 ± 5.24
*t*	9.949	10.624	5.183	12.145
*P* value	<0.001	<0.001	<0.001	<0.001

**Table 2:** Comparison of changes in serum SCC-Ag, CA125, and CEA levels before and after treatment (mean ± SD).

Time	SCC-Ag (ng/ml)	CA125 (U/ml)	CEA (ng/ml)
Before treatment	10.26 ± 0.85	48.19 ± 6.58	14.28 ± 2.16
After treatment	6.82 ± 0.72	40.52 ± 6.18	9.74 ± 2.07
*t*	20.250	5.572	9.951
*P* value	<0.001	<0.001	<0.001

**Table 3:** Comparison of area, standard error, progressive significance, and 95% confidence interval of various indicators.

Test result variable	Area	Standard error^a^	Progressive significance^b^	95% confidence interval	
				Lower limit	Upper limit
SCC-Ag	0.721	0.056	0.000	0.611	0.831
CA125	0.721	0.056	0.000	0.611	0.831
CEA	0.686	0.058	0.003	0.572	0.800
Colposcope	0.860	0.043	0.000	0.775	0.946
Combined test	0.930	0.032	0.000	0.868	0.993

^a^Nonparametric hypothesis; ^b^null hypothesis, real area = 0.5.

**Table 4:** Comparison of positive rate, sensitivity, and specificity of various indicators.

Indicator	SCC-Ag	CA125	CEA	Colposcope	Combined test
Positive (cases)	34	38	36	37	41
Positive rate (%)	39.53	44.19	41.86	43.02	47.67
Sensitivity (%)	74.14	69.35	68.25	87.76	91.49
Specificity (%)	82.69	89.58	86.00	87.76	95.56

## Data Availability

Data that support the findings of this study are available on reasonable request from the corresponding author.
